# Short History of Epidemiology for Noninfectious Diseases in Japan. Part 2: Epidemiology of Stroke and Hypertension up to 1970

**DOI:** 10.2188/jea.18.2

**Published:** 2008-02-28

**Authors:** Kunio Aoki

**Affiliations:** 1 Professor Emeritus, Nagoya University, and Honorary President, Aichi Cancer Center; 2 Chairman, The Nagoya City Council of Social Welfare

## INTRODUCTION

The mortality due to stroke had been around 170 per 100,000 population in 1930s in Japan, ranking second only to that from tuberculosis. According to Aizawa, only a few studies were reported on the topic of stroke at scientific meetings of internal medicine. In those days, it was only an occasional topic of discussion among the clinical specialists at the cerebrovascular sections of the Japanese Society of Circulation and Neurological Society, indicating a paucity of general interest in this disease entity.^1^ This was an era when average life expectancy was only slightly over 40 years; mortality for young and middle-aged adults was very high; the middle-aged and aged population rarely visited medical institutions; diseases were often diagnosed after they had advanced to a grave state; and effective therapies were relatively rare. Sasaki stated that in the 1930s, few medical researchers focused on the mortality from cerebral apoplexy and did not note its seriousness until Sadamu Watanabe called their attention to it.^2^^,^^3^

Under the war-time regime, the Ministry of Health and Welfare was newly established. The medical insurance system was reinforced and a health screening system was initiated at some work places. Subsequently, more patients sought medical attention for stroke and the significance of this illness began to be recognized. The Life Insurance Association had initiated a study on stroke. Realizing the significance of this trend, it offered a research fund amounting to 30,000 yen for a period of 3 years to the Japan Society for the Promotion of Science to organize a research group. The proposal was accepted and in April 1941, the 43rd Subcommittee for the study of Prevention of Cerebral Apoplexy of the Japan Society for the Promotion of Science was organized. This subcommittee was composed of the following 12 members, with the selection of Professor Chujiro Nishino as its Committee Chairperson: Tsugitake Isshiki, Chuji Ito, Seizo Katsunuma, Otoya Kimura, Yasutoshi Furuse, Takayuki Sasa, Takao Takada, Chujiro Nishino, Goichi Hiramitsu, Shunichi Mashita, Terasu Mozai, and Sadamu Watanabe. It was reported that this research group was scheduled to conduct discussions while representing both universities and insurance companies. They deliberated on the results from statistical, clinical and histopathological studies, collected pertinent literature, and while placing particular emphasis on the relationship between blood pressure and cerebral apoplexy, delved into topics that included specific factors (i.e., risk factors, heredity, specific somatotypes, environmental elements, prevention and measures to reduce the mortality of stroke).^4^^,^^5^ One may get an impression this the academic study on stroke was initiated in a somewhat unorthodox manner in Japan.

During this period, the word epidemiology was rarely used in relation to non-infectious chronic diseases. In the category of circulatory diseases, Naosuke Sasaki published a study in 1952 entitled “Epidemiology of Apoplexy” and in 1958, the 29th Congress of the Japanese Society for Hygiene hosted a symposium that focused on the epidemiology of hypertension. In the following year, at the 16th Symposium on Hypertension (chairperson, Fusakichi Nakazawa) of the Japanese Medical Association, Noboru Kimura and Katsuya Itahara presented a speech entitled “An Epidemiologic Study of Hypertension”. It appears that those researchers who returned from studies in the United States began to start using the word epidemiology then. With a grant from the Ministry of Education, a group led by Shigeo Okinaka started a study in 1962 and published “An Epidemiologic Study of Apoplexy.” The 1966 ABCC (Atomic Bomb Casualty Commission) Symposium held in Hiroshima selected “Epidemiology of Coronary Artery Diseases and Apoplexy” as its major theme. It appears that the word epidemiology was firmly established in Japan in the 1960s; therefore a historical overview of epidemiologic studies on apoplexy and hypertension up to the 1960s must include those represented by topics such as hygienic and public health studies, statistical observations, regional or geographical studies, surveys on population groups and etiological investigations. After the 1970s, studies on circulatory diseases made dramatic progress: the current review encompasses the activities up to this period. However, it is very difficult to include all the literature published since 1940 so the current efforts have focused on the description and evaluation of representative studies related to epidemiology. The author awaits further evaluation on this topic.

## STATISTICAL STUDIES ON APOPLEXY BEFORE 1941 CONDUCTED BY PHYSICIANS EMPLOYED BY LIFE INSURANCE COMPANIES

This overview had originally been intended to start by covering the period immediately after the end of the World War II. However, it was found that as early as the 1910s, those physicians employed by life insurance companies on their own initiative conducted their studies on mortality from apoplexy and blood pressure at various population levels, which occupied an important position in our studies. Thus the current review starts by introducing these efforts.

As well-known, the mortality of the subscribers to life insurance policies has a very important bearing on the operation of insurance companies. Thus surveys had been conducted from the early days on the health status of subscribers and new applicants. There was a wealth of international information on this topic: it appears that these companies utilized new statistical techniques and operated their business based on the probability theory. Consequently, apoplexy, with its high mortality among the middle-aged and aged population, was an important topic to these companies. They employed physicians on full-time basis, adopted the blood pressure determination procedure early, and instituted a deferment system for those applicants with high blood pressure. Follow-up studies were conducted on the subscribers in an attempt to detect the risk for early death. Around this time, a Society of Life Insurance Medicine was organized through which studies related to apoplexy and blood pressure were continued. In the 1930s, in particular, an increasing number of studies came to be published. Notable among these studies was a comprehensive analysis by Kurosawa of 1938.^6^ He compiled the data on 1,293,755 subscribers of life insurance of various companies for a 20-year period between 1912 and 1931 and conducted a statistical evaluation of 1,445 who succumbed to apoplexy to analyze the relationship between the factors related to their surroundings and body constitutions. The mortality from apoplexy was about 10% of the total mortality during this period and the mortality from apoplexy among the subscribers to life insurance showed tendencies for an increase for both sexes during this 20-year period. When classified by age, the mortality began to increase in the 40s, followed by rapid increases as the patients aged: it was high in cities and low in agricultural villages. When classified by occupations, the mortality was high among those engaged in home industry and free-lance workers between the ages of 46 and 55 years, while it was low among farmers of the same age groups. Among those in 36 to 70 years who succumbed to apoplexy, the attacks occurred frequently while the patients were defecating, experiencing physical labor, having a meeting, dining, bathing or drinking alcoholic beverages. A large number of women suffered attacks while bathing. Attacks also occurred often in the morning and evening and more often in winter than in summer. Familial clustering was observed, and notable body type among the victims was small stature and large girth and body weight. It was concluded that the risk for apoplexy is greater among those with a relatively good nutritional status and satisfactory physique.

There was an excellent study on the blood pressure (normal blood pressure and its range) of those with a life insurance policy.^7^ In 1939, Tsugitake Isshiki et al^8^ compiled studies by physicians employed by insurance companies and described such details as the history of the use of blood pressure as a diagnostic means, its determination, the relationship between blood pressure distribution and age-specific characteristics, body compositions, occupations, heredity and racial stock, blood pressure and prognosis, mortality. A comparison with data from the United States was done, too. For a statistical observation of blood pressure, blood pressure determination was practiced at the time once applied for life insurance in Japan (as early as in the 1920s) as a method to determine one’s health condition. In 1939, 3 insurance companies jointly conducted a follow-up study on 1,301 individuals over 30 years who were refused subscription for such reasons as a high blood pressure. Subsequently, they reported that in spite of their relatively young age, the risk for stroke of those with a systolic pressure over 160 mmHg was more than twice that of normal individuals. They discussed the so-called normal level of blood pressure and suggested a systolic pressure of 159 mmHg as normal for an average person. In the same year, Isshiki published a study entitled “Statistical Observation of Cerebral Apoplexy”^9^ and investigated the clinical status at the onset and following stroke, as well as the relationship between blood pressure at the time of applying for insurance and onset of cerebral apoplexy in 4,759 life insurance policy holders who succumbed to the disease between 1939 and 1943. Stroke occurred in 258 and the mean blood pressure within 6 months of the attack was very high (191.76 mmHg for men and 190.43mmHg for women). The blood pressure reading was below 149 mmHg only in 7.5% of men and 4.1% of women. The close relationship between blood pressure and prognosis was discussed. Already by 1937, Sadamu Watanabe evaluated mortality among obese individuals.^10^ He conducted a 9-year followed-up study on 834 policy holders who were 30 to 49% overweight over normal individuals and reported that among these obese persons, mortalities from nephritis, cerebral apoplexy and circulatory diseases were high: he stated that at ages over 45 years, in particular, the risk for death due to cerebral apoplexy rose rapidly. However, compared with the data from the United States, this risk was still low. He pointed out, on the other hand, that the mortality due to tuberculosis, which was rampant in those days in Japan, was very low in those obese individuals. He stated that a survey such as this was needed every 5 years.

Recognizing a need for getting accurate statistics on the incidence of stroke in Japan, Sadamu Watanabe in 1940 compared the mortality in Japan with those of European countries. He discussed the mortality due to stroke was decidedly higher in Japan than those of other countries and when confined to domestic figures, the mortality was higher in the Tohoku Area, in particular in Akita Prefecture. He also discussed alcohol drinking as an etiological factor.^2^ There are many other reports on this subject and one should note that many were based on long-term observations.

In 1905, a new manometer by indirect blood pressure reading was introduced by Korotkoff. This new type of manometer was introduced to Japan shortly thereafter and by the 1910s at the latest, it was widely used on clinical patients. The life insurance companies in Japan started statistical analyses of blood pressure in the 1920s. In the 1930s, basic data on blood pressure distribution among Japanese became available for both genders and all age levels.

The statistics collected by life insurance companies might be marred by bias because the subjects were limited to voluntary participants and life insurance policy holders. However, their observations covered a large portion of the population (exceeding 10,000) and their epidemiologic coverage across the sexes and age groups seemed fairly accurately be reflected the actual status of the Japanese population at that time. These statistics were also helpful in making comparisons among various regions in Japan. Therefore in an age when the etiology of stroke was still unknown, the relationship between the above-mentioned frequency distribution and blood pressure and the basic results concerning its etiology were information amply contributory to the study of the general population in this country. However, at medical congresses that were hosted by the leaders of medical universities, there were very few records on the introduction or evaluation of these studies.

### Studies by the Nishino Group, in Particular, Epidemiologic Studies^5^

Several studies were initiated by the Nishino Group, which was organized in 1941.

Since 1941, research conferences by this Group have been held 2 to 3 times annually or altogether, 14 times by January 1947 after the Second World War. In March 1948, Dr. Chujiro Nishino, the chairperson of the group, edited the summaries introduced at these conferences and he published them with superb efforts in 1950 in spite of what appeared to be insurmountable difficulties after the war.

The members of the group in 1945 consisted of professors at universities (Hokkaido University, Tohoku University, Tokyo University, Keio University, Chiba University, Niigata Medical University, Nagoya University, Kyoto University, Osaka University, Keijo University, and Manchu Medical University), physicians affiliated with life insurance associations (Teikoku Seimei, Nihon Seimei, Meiji Seimei, Chiyoda Seimei, and Daiichi Seimei), and representatives of the Life Insurance Institute and Japanese Medical Academy. The postwar committee meetings were still at the stage in which their prewar activities were being compiled. In addition to clinicopathology of stroke patients, a summary of pathological studies that had been conducted, etiology and the involvement of genetic factors, the correlation with patients’ life styles and the disease onset were reported. Among these topics, the current study focuses on the etiological and epidemiologic studies.

### Etiological Studies

In the area of public health, Shoji Kondo et al^11^ compared stroke mortality among prefectures and indicated that it was higher in the Tohoku area, in particular in Akita Prefecture, lower in the San-in area and that in the former, mortality was higher even among those still between 20 to 50 years of age. Furthermore, they conducted a survey that included interviews in 14 prefectures and 108 villages and reported that stroke mortality varied widely among areas. They also referred to area-related gaps in age distribution in stroke mortality. Those areas where stroke mortality was higher were characterized by a frigid climate; the inhabitants engaged mainly in rice cultivation and consumed a large quantity of rice, table salt and alcoholic beverages, all of which correlated positively with stroke mortality. On the other hand, among those engaged in the fishing or dairying occupations, stroke mortality was lower. The mortality for men and women varied by area and a relationship between stroke mortality and areas with high and low life expectancies was suggested. The stroke mortality showed familial clustering, which suggested an endogenous cause for the disease; but the involvement of an individual’s life style was still considered more important in the etiology. In part of these surveys, blood pressure was recorded and followed up for hypertensive patients -- those with a blood pressure over 130 mmHg or age plus 90 mmHg -- and an attempt was made to set an abnormal blood pressure level from the observations. For clinical epidemiologic study, Imasato Donomae et al,^12^ over a period of 3 years starting in 1939, investigated the clinicopathology and geographic distribution of 8,891 patients who succumbed to stroke in Chiba Prefecture. In addition, they conducted a population survey on 10,372 individuals residing in 3 agricultural villages in 1943 and reported the results. In this group, the so-called brain death occurred in 170 to 220 per 100,000 population (an average for 10 years). Like in the study by Shoji Kondo, regional differences within the prefecture and familial clustering were noted in the frequency of occurrence. In the villages showing higher stroke mortality, the mean blood pressure recorded at a mass examination was higher and the incidence was related to heavy alcohol consumption as well. These researchers also discussed prognostic factors. In addition, they reported on the physiopathology of hypertension and the implications of arteriosclerosis, cerebral hemorrhage and blood pressure of the ophthalmic artery.

Taro Horimi et al^13^ of Osaka University measured the blood pressure of 5,500 ambulatory patients between 1941 and 1944 and evaluated its distribution, annual fluctuations and relationship with the quantities of food consumed, meat or vegetable consumption, alcohol drinking and smoking. They also pointed out that the patients’ blood pressure was reduced during the war.

Seizo Katsunuma (Nagoya University)^14^ noted that they found abnormalities in the walls of the cerebral arteries in autopsy studies of patients who had had normal blood pressure prior to the attack but still succumbed to cerebral apoplexy. They subsequently attempted to determine the blood pressure in the head region (superficial temporal artery) and cited the significance of measuring ocular tension. They determined the blood pressure of 2,029 healthy individuals in Nagoya and discussed circadian variations in pressure, its changes due to shifting postures and relationship to the pressure in the superficial cerebral artery. This researcher introduced the results of his blood pressure survey conducted in Taiwan and the Goto Islands of Kyushu. He found that the blood pressure of the residents of those islands was lower than that of Japanese living on the main islands and placed significance on the consumption of a large quantity of seaweed on Goto Island in the low incidence of stroke. This is considered to be a form of mass survey on the clinical physiopathology of stroke at that time.

In clinical areas, the clinical physiopathology of stroke was investigated, including the psychiatric aspect of the patients; but it was omitted in the current study.

For clinical studies, Isshiki^15^ presented an overview on the statistics related to stroke and hypertension among life insurance policy holders. First, he stated that compared with the statistics from the United States, the blood pressures of Japanese were not at all low, rather it was higher. His review included the contents of 16 presentations by physicians affiliated with life insurance companies. From these statistics, Isshiki stated that 31.1% of men and 28.0% of women between the ages of 50 to 60 years exhibited systolic pressures exceeding 150 mmHg; and that although there were some variations among reports, a marked regional difference and those related to occupations and dietary habits existed. Furthermore he comprehensively reported on topics such as the relationship between body frame and blood pressure, blood pressure of Taiwanese, blood pressure and mortality, apoplectic attack and blood pressure, heredity, alcohol drinking, smoking and the physiopathology and contributing factors of an apoplectic attack. He introduced a study on blood pressure distribution among female policy holders of life insurance in Japan, a work by the late Jiro Hiyoshi, and discussed age- and gender-stratified blood pressure distribution. He also examined the correlation of somatotypes with blood pressure and percentage of hypertensives among life insurance policy holders (54,081 men and 13,305 women) between 1932 and 1934, as well as blood pressure distribution among 33,250 women between 1935 and 1940. For those over 55 years of age, he reported that more than half of them showed a blood pressure over 140 mmHg. He also reported on the relationship between blood pressure and apoplectic attack. He stated that compared with the Taiwanese, the blood pressure of Japanese was higher; however, after 1943 it was lower because of a shortage of food supply due to the war.

Takao Takada et al^17^ described the age-stratified blood pressure distribution among life insurance policy holders (63,000 men and 7,000 women) in 1939 and 1940 and showed statistical evidence that blood pressure was lowered with reductions in body weight during the war. The blood pressure was found to be higher in agricultural villages, in particular in the north of the Kanto area, Hokuriku and Kyushu with a notably high incidence of hypertension in Akita Prefecture. When stratified by occupations among the population over 40 years, the incidence was higher among those engaged in agriculture and fishing and among laborers. In general, weight gains were associated with a higher blood pressure. The relationships between blood pressure readings and physical conditions and similarities in life style among patients’ families were described. Sadamu Watanabe^18^ divided the subjects studied described by Takada, et al. by 5 years and evaluated changes in blood pressure as one ages. He also reported that hypertensive patients were exposed to high risks of apoplexy, circulatory diseases and kidney diseases but unlikely to succumb to tuberculosis and that the incidences of alcoholism, cardiac diseases and abnormal urinalysis results are higher in stroke patients. Masashige Nakagawa et al^19^ reported that blood pressure was lower among female divers and fishermen and their families, suggesting a relationship between blood pressure and seaweed consumption. Studies were conducted on such topics as body types of stroke patients, the relationship between parity and stroke in females, clinical conditions of apoplectic attacks, their causative factors, complications and changes in blood pressure. There is a report describing high incidences of liver cirrhosis, diabetes mellitus, tuberculosis and pneumonia among alcohol drinkers.^20^ Shoji Mishina et al^21^ reported on the cause of death due to stroke: they noted the correlation of stroke mortality with gender, age, season, body types, heredity, alcohol drinking, psychological stress, overwork and exposure to cold and extreme heat but they did not recognize a relationship with smoking. Itazawa^22^ conducted a statistical analysis on the difference of body types of patients who succumbed to tuberculosis and stroke. Surveys were also conducted on the distribution of blood pressure in Taiwan and the Korean Peninsula.

Sadamu Watanabe^23^ introduced the results of his study on changes in the incidence of stroke during and after the war. He stated that stroke mortality had been increasing annually since 1935, began to decline somewhat after 1941, the mortality in 1947 (after the end of the Second World War), in comparison with 1935, was reduced by 25% across all ages, by 50% between the ages of 45 and 54 years, and by about 38% between 55 and 59 years. Around this time, the aged population increased by 10 million: it was surmised that the actual reduction in death from stroke was considerable. For the reasons for this change, a drop in blood pressure due to a scarcity of food and in particular, reductions in the number of hypertensive patients were cited. This phenomenon of lowered blood pressure was observed in every prefecture but it was most prominent in Tokyo. The distribution of blood pressure among the population did not change much in the next year (1948) but Watanabe feared that the old pattern would return in the future.

Surveys conducted by physicians affiliated with life insurance companies have a history going back some 20 years. These were statistical observations involving large cohorts (in units of 100 thousand or 1 million subjects); consequently there was a large impact on the studies on cerebral apoplexy. The criterion for an abnormal systolic blood pressure was variously set -- e.g., 130, 140 and 150 mmHg. After the war, then, the criteria that had been used until recently in Japan -- 140 mmHg for systolic pressure and 90mmHg for diastolic pressure -- were adopted. However, it does not appear that these criteria were necessarily used everywhere. The common belief was that those with life insurance were relatively well-to-do and healthier and they did not necessarily represent the general population. Another comment was that medical history taking and examination for qualifying for life insurance were conducted by a very large number of affiliated physicians located throughout the country, and methods or diagnostic criteria often lacked uniformity and the practice lacked objectivity. There were few treatises by university-affiliated researchers who actively evaluated and utilize these data.

For the conclusion of this report, Nishino made the following statement:

Taking consideration into diagnostic accuracy at that time, it is certain that the mortality from a stroke and related diseases among Japanese is far higher than among the western population. A non-hygienic environment and unhealthy life style in this country may have considerable significance in explaining this discrepancy. The relationship with body types and other elements may be mediated by hereditary and congenital factors; but those factors that are acquired later in life may have far more significance. It is certain that the frequency of the development of diseases such as hypertension, nephrosclerosis and cerebral arteriosclerosis parallels the incidence of hypertension. In autopsy observations, stroke does not develop without rupture of the cerebral arteries: hypertension, together with cardiac hypertrophy and renal atrophy, are considered to be a stage preliminary to the development of stroke. In such an instance, cerebral softening and hemorrhage occur intertwined with the development of stroke. Miliary aneurysm may also develop. Many instances of subarachnoid hemorrhage are secondary to stroke.

Hypertension occurs frequently in mountainous regions, but its incidence is low in the coastal areas. Consumption of a large amount of seaweed in the coastal region appears to give some hint to its relationship with the development of stroke. Alcohol drinking, especially the drinking of poor quality rice wine (*sake*) increases the incidence of stroke. For its etiology, evaluation of the alcoholic component deserves some investigation.

Experimental studies found that cerebral circulation is eminently affected by abnormal circulation in other organs, especially the kidney. The results of clinical investigations provided many facts that could trigger a stroke and yielded new findings that would aid prognosis or emergency treatment.

The major thrust in stroke prevention is in early treatment and prophylaxis of hypertension, nephrosclerosis, cerebral arteriosclerosis and other diseases that are considered to be in the background of this major catastrophe. Furthermore, the life style that may trigger an apoplectic attack, such as sudden changes in blood pressure, should be avoided as much as possible. Nishino concluded his report by stating that hygienic standards for the environment must be improved and the general public must be educated so that their life style will be hygienically ameliorated for the purpose of preventive medicine.

His was a valuable report that indicated a direction for future research and health policies and that deserved respect in the field.

## EPIDEMIOLOGIC STUDIES ON STROKE AND HYPERTENSION AFTER THE SECOND WORLD WAR

During the period between 1945 and 1950, Japan lacked supplies of food, clothes and housing. Jobs were scarce and people had to make best efforts to maintain a minimal level of daily living. The mortality from acute infectious diseases, which constantly frightened people, was reduced in 1947 when the people’s diet gradually began to improve. Mean life expectancy also began to be prolonged with the middle-aged and aged population segments continuing to increase, resulting in a noticeable rise in mortality due to stroke after 1956. Perhaps due to a lack of knowledge, effective policies were slow in coming. While under the administration of the occupying forces, a higher priority was placed on building basic organizations to combat such problems as the control of acute infectious diseases, maternal and infant health care and regional public health. The countermeasures of tuberculosis patients who numbered over 3 million at that time, was another urgent problem. Thus the government did not have the resources to look into chronic diseases that affect the middle-aged and aged population.

In 1947, when the publication of vital statistics was resumed for the first time after the war, the total mortality for that year was low (14.6 per 1,000), comparable with the figure of around 16 for 1941-42. The total mortality rate, which continued to decline gradually thereafter, was explained mainly by a drastic reduction in the mortality due to undernutrition and communicable diseases including tuberculosis. Mortality from pneumonia continued to decline (around 150 per 100,000 population in the first half of 1940, 130 in 1947 and 66.2 in 1948). The decline in mortality due to cerebrovascular diseases was still observed in 1948 (117.9 per 100,000), which however began to climb later.

The Japanese population over the age of 40 was about 2 million in 1950, but it increased rapidly thereafter. The mortality due to cerebrovascular diseases was 136.1 per 100,000 people in 1955 but rapidly rose to 160.7 in 1960 and 175.8 in 1965, returning to the level of the 1930s. In 1960, the number of people who succumbed to these diseases exceeded 160,000. The mortality from hypertensive diseases, a newly created category in 1950, gradually rose from 11.9 per 100,000 to 19.3 (about 20,000 victims) in 1965, indicating that the mortality from cerebrovascular diseases exceeded the corresponding figure before World War II.^24^ The age-stratified distribution of the deceased indicated that many families were losing their main support, and a serious effect on the socioeconomics attracted public attention. Meanwhile heart disease-related mortality was still low, exhibiting a pattern different from those in the western world.

With this background, an increasing number of medical researchers began to engage in descriptive epidemiology on stroke and heart diseases using mortality statistics in the 1950s, describing the magnitude and urgency of the problems. Financial support by the government was very small but those medical researchers with a sense of crisis formed an alliance with local legislative bodies and engaged in surveys to assess the actual status of hypertension and mortality due to stroke in specific population group. This practice then gradually spread throughout the country. In addition to clinico-pathological states, they pursued the correlation between the onset of these diseases and climate, air temperature, soil composition, the water supply, occupations, and serum cholesterol levels, which might have given a key to preventing these diseases.

In 1952 the Peace Treaty was signed and Japan recovered her sovereignty. Starting in the following year, the Japanese Government was able to prepare independent annual budget. For the public policies concerning disease control, a large budget was prepared and approved to conduct a nationwide survey on the prevalence of tuberculosis patients, which was then becoming a serious social problem. Some funds were allocated to conduct surveys on hospitalized cancer patients but surveys on stroke patients were postponed.

Here a research survey that was conducted from around 1950 in the Tohoku region, with a traditionally high stroke mortality, was introduced in details to represent those projects conducted throughout the country at that time. The study had been conducted energetically on stroke mortality and hypertension on a resident basis. Furthermore the history of the study of circulatory disease care by industrial organizations was reviewed and the studies on early stage stroke and hypertension, presented at academic meetings, were concisely described.

## STUDIES ON STROKE CONDUCTED IN THE TOHOKU REGION IN THE 1950s

In the Tohoku region, known for high stroke mortality, Fusakichi Nakazawa (Internal Medicine, Tohoku University)^25^ and Tokuro Fukuda (Internal Medicine, Chiba University)^26^ conducted clinical epidemiologic surveys on stroke as preliminary steps for more precise studies to be conducted there. Fukuda noted a high frequency of hypertension among the farmers of Akita Prefecture. He also recognized a high intake of salt by the area residents and based on urinary chlorine content, estimated this intake to be 26.3 grams per day. However, it is said that he did not recognize the relationship between salt intake and hypertension. It has been recorded that the Public Health Department of Akita Prefecture and Yuji Kudo (Iwate University) started taking blood pressure measurements among the residents in 1952.

Between 1954 and 1955, Eiji Takahashi, Naosuke Sasaki (Hirosaki University) and others initiated a survey among the inhabitants of Aomori and Akita Prefectures,^27^^-^^35^ which included blood pressure determination. Takahashi and Sasaki exhibited in figures stroke mortality that increased, starting around middle-age, with marked regional differences. To explain these epidemiologic findings, they investigated the relationship between the blood pressure distribution and the life styles of local residents, the ultimate goal being to find potential stroke patients while still in the early stage. Nationwide stroke mortality rapidly rose at the age of 50. In Akita Prefecture, the blood pressure of residents began to rise while they were in their 40s and became twice the figures of other prefectural residents who were between 55 and 59 years of age; and within this prefecture, there was a gap in mortalities between towns and villages. They compared these results with the mortalities of others areas in Japan and found that the Tohoku region as a whole was associated with high stroke mortality while that of western Japan was low. In particular, the stroke mortality was low in the Shikoku region, where it was only one-half that of the Tohoku region for those between 30 to 59 years. They also compared stroke mortality in cities, towns and villages of Akita, Aomori, and Okayama Prefectures and found that it was already high at a relatively young age in Akita Prefecture. It had already been reported that in the Hirosaki area, the blood pressure of life insurance policy holders was also higher than the national level. In other words, stroke mortality was area-specific, leading to a theory that there probably were specific etiological factors. Takahashi continued epidemiologic studies on stroke in Miyagi Prefecture while Sasaki conducted similar studies in Aomori and Akita Prefectures.

For etiological factors they suspected the impact of low environmental temperatures and the presence (or absence) of indoor heating. The low stroke mortality in frigid Hokkaido led to a suspicion that the prevalence of indoor heating might be involved in this etiology. Takahashi observed that blood pressure rose in the same person in winter and that a relationship existed among room temperature, income level and stroke mortality. Fishermen engaged in inshore fishing were employed in the cultivation of oysters and seaweed in winter, leading to their exposure to cold seawater. Thus it was believed that they experienced a rise in blood pressure. On the other hand, the low blood pressure of miners working underground was explained by the high humidity and high temperature underground. The diet of those miners working underground did not differ from that of those working above ground. Takahashi studied deep sea fishermen, whose blood pressure was high in the summer and explained this by their busy schedules during the summer, citing a relationship between blood pressure and labor conditions.

As for the implication of diet, stroke mortality was positively correlated with rice, negatively correlated with consumption of meat, milk, egg and the like. In Miyagi Prefecture, consumption of a large quantity of rice was correlated with high blood pressure; and the intake of various types of vitamins was negatively correlated. It was reported that rice yield was related to obesity in those of younger age. Sasaki also presented similar data.

Sasaki, who considered salt consumption to be an important factor in the study of stroke mortality, developed an empirical study. He clarified the correlation between the consumption of pickled vegetables (a source of dietary salt) and hypertension. On the other hand, the scarcity of vegetable patches was inversely related to the blood pressures of family members, which led to the impact of vegetable consumption. He also noted the low mean blood pressure among those working in apple orchards, in comparison with that of farmers, he began to investigate the possible effects from consuming a large quantity of apples. To test the hypothesis that potassium contained in apples inhibit the actions of salt, Sasaki conducted a pre-clinical experiment to substantiate the correlation between the two, and be pointed out the workers in apple orchards were relatively high socio-economic status than that of the farmers. The diets of those in farming villages with known high blood pressures were compared to those in a fishing village with low blood pressures. The consumption of greater quantities of proteins, fat, vitamins, calcium and iron was related to the low blood pressures of the residents of fishing villages.

Sasaki also conducted a detailed survey on the consumption of miso soup, pickles and soy sauce as sources of salt and established that the amount of *miso* soup consumed is related to blood pressure; in particular, it was found that the salt content of *miso* soup was an important factor. Sasaki divided the country into 11 districts; and by using the data from a nutrition survey among farmers (conducted by the Ministry of Agriculture), a statistical correlation analysis was conducted on the nutrient intake and incidences of stroke among farmers. It was found that stroke mortality was higher in those areas known for high consumption of foods containing salt, niacin, vegetable proteins and iron or in areas where the people have had a large total quantity of vegetables, pickles, and food of vegetable origin and seasoning. Among the seasonings, *miso* was regarded to be a risk factor, while vinegar was believed to inhibit the development of hypertension. The relationship between consumption of a large quantity of white rice and stroke, as proposed by Kondo, was no longer statistically valid and rice consumption was regarded to be indirectly related. Food with the prophylactic factors such as seaweed, soybeans and carrots, the effects of which had already been reported, contain a large quantity of potassium and were found to be consistent with Sasaki’s findings. He stated that intake of a large quantity of salt is often closely related to heavy labor and the etiology of stroke must be pursued with complex interactions of several factors, such as life style and type of work. The results of these studies were introduced overseas by Takahashi^35^ and attracted much attention.

Sasaki in 1976 analyzed stroke mortality in several cohorts stratified by birth years since the Meiji Era. He stated that the mortality had hardly changed before 1945: it decreased during and after World War II. The rate rose again in 1953. Since 1965, however, the overall mortality due to cerebrovascular diseases began to decrease among men due to reductions in mortality from cerebral hemorrhage, while among women, stroke mortality continued to decline after the war. It was suggested that the background factors responsible for the development of cerebrovascular diseases in women were diminishing even before the war. The reason is not clarified yet. Sasaki also confirmed that in the areas where stroke mortality was known to be high, the blood pressure distribution among youths was already at levels higher than in those areas where the mortality was known to be low and it continued to rise even higher as they grew older. He suggested that stroke management should be started while the subjects were still in their teens.^32^^-^^34^

The high incidence of stroke could not be explained by high salt intake in some areas: localized areas with high mortality were scattered throughout the country. To explain factors that were area-specific, surveys were scheduled in various areas. Such moves were reflected in the reports introduced at academic meetings (described later). Perhaps giving due consideration to this background, the Ministry of Health and Welfare added blood pressure determination to the National Nutritional Survey conducted in 1956. These national data were later found to be very valuable basic material.

## POSTWAR ACTIVITIES OF PHYSICIANS ASSOCIATED WITH LIFE INSURANCE COMPANIES

Studies on blood pressure and stroke by physicians affiliated with life insurance companies were continued into the postwar period.^36^^-^^41^ According to a statistical observation of a series of blood pressure readings by Kiyoshi Kanai,^39^ 150 mmHg had been adopted for the normal systolic pressure. In 1957, Shoji Hirao introduced the results of a statistical study on the blood pressure of Japanese,^41^ in which he stated that blood pressure during the developmental stage shows a simple normal distribution; but after the maturation period it shows a complex of blood pressure groups composed of 2 types of normal distribution. The blood pressure determination may be expressed by a rejection ellipse but it may differ in each age group. The normal threshold of blood pressure has been set at 140 mmHg and 90 mmHg for systolic and diastolic pressures, respectively.

Mitani et al^42^ and Isshiki et al^43^ reported on the relationships among blood pressure, prognosis and cause of death. Torii et al^44^ and Mishina et al^45^ conducted follow-up studies on 27,602 individuals who were turned down after their physical examination for life insurance because of hypertension and concluded that in comparison with the general population, the mortalities from cerebral hemorrhage, cardiac disease, and renal disease were 3.5, 1.6, and 1.7 times higher in these individuals. There were also a number of other excellent studies.

## MANAGEMENT OF CIRCULATORY DISEASES AT INDUSTRIAL ENTERPRISES

To combat tuberculosis that was rampant in those days, many leading enterprises in this country embarked on tuberculosis screening and patient care. These programs began to show positive results and tuberculosis management became well-organized around 1960, with the threat of this disease fading away from people’s minds. Thus industrial physicians began to contend with another health problem. Having gained confidence by a success in tuberculosis control, they began to focus their attention on the rapid increase in the incidence of stroke among those in the prime of their lives. Industrial enterprises also regarded this as a serious problem involving their employees and began to plan preventive measures. The time was ripe because the close relationship between hypertension and stroke had already been established and blood pressure measurements were readily conducted on groups of employees.

Fusao Akiyama was originally a clinician, but had been interested in preventive medicine. He insisted that the age when a plan for stroke prevention was abandoned simply because its cause was not known was already over: instead causative factors should be investigated through epidemiologic studies and active preventive measures should be taken. In particular, he studied the natural history of hypertension and established the following hypothesis: multiple factors operate in a complex manner in the development of a stroke, which occurs as a result of many years of a smoldering process: the prognosis of such a disease that develops with a putative background of many years standing, if discovered early enough, can be sufficiently improved by medical treatment and health management. To implement early detection and patient care, Akiyama envisioned a system that would provide care with a slant toward social medicine. Because screening and management of the disease must be conducted over an extended period, he believed and was actively involved in the campaign with industrial enterprises and area residents in 1953.^46^^,^^47^ He was strongly impressed by the Framingham study that was started in the United States in 1947, an epidemiologic study on arteriosclerosis and hypertensive heart diseases among residents of a small area that was conducted through their health management and implementation of health policies. Since then, he studied other similar long-term studies that were conducted in a few areas, also in the United States and planned for studies and prevention programs for stroke and hypertension that could be implemented in Japan.

Specific hypertension control programs included the following: first, a simple screening procedure to single out those with abnormal blood pressure; at a second examination, these patients would be subjected to detailed analyses, with some follow-up regularly thereafter; and the third, preventive measures offer such procedures as avoidance of exacerbation, early treatment and aftercare of the condition. Akiyama also studied the possibility of introducing electrocardiography to fortify the screening procedure but considerable expense was inevitable in such a step.

The screening methods have been studied by many researchers since then. Innovations were made to incorporate medical history taking, measurement of body height and weight, physical examinations, urinalysis, blood pressure determination, electrocardiography and fundus examination so that many individuals could be examined in a short time. It was intended that the early diagnosis of arteriosclerosis and risk factors for stroke would be evaluated through repeated periodic examinations and obtaining base values for blood pressure and its sequential changes from data in a time sequence, the results of which might lead to prevention of stroke.^48^^-^^50^

The control of circulatory diseases based mainly on the measurement of blood pressure was considered to be rational and efficient at organizations such as industrial enterprises that had company systems and budgets at their disposal. At the work place, circulatory diseases are not infectious and do not require a social defense (as with tuberculosis) so a business organization had no responsibility. However, in view of unnerving reports by the mass media about the sudden deaths of managers in responsible positions, the effects of which affected not only production but also the morale of employees. Improvement in one’s health status by health examinations and providing education, which ultimately brought considerable advantages to conducting their operations, these business enterprises appeared to have come around to approve these suggestions on circulatory care. At that time the labor and management relationship was somewhat delicate, which also explains the latter being willing to allocate a portion of their budget to these projects. In a short while, circulatory care at the work place was initiated by many corporate bodies, while industrial physicians engaged in active information exchange and held research and discussion meetings among themselves.^51^^,^^52^

For a general trend, a policy to combat stroke and hypertension for regional inhabitants was started in agricultural villages in the Tohoku region, then spread to other villages throughout the country. In large cities, screening mainly of employees in industrial concerns and studies on preventive care were started, then spread to the rest of the country. The concept of screening, management and prevention was highly attractive to villages that were plagued by a physician shortage. Screening teams including researchers were organized not only in the Tohoku region but also in a number of areas throughout the country, i.e., Kanto, Nagano, Yamanashi, Aichi, Mie, Kyoto, Osaka, Yamaguchi, Nagasaki, and Kyushu. They engaged in an annual screening, followed by appropriate health care. This move was related to an increase in the income of people in agricultural regions after the war and changes in farmers’ attitudes towards health. Through the care of circulatory diseases, the Association of Agricultural Medicine and Rural Health achieved notable development.

Meanwhile, the health screening format that was initiated in the United States (aptly called a “human dock” in Japan) was originally intended for the prevention of circulatory diseases; but the urban population later became interested and clinical physicians actively initiated the service at hospitals. The practice spread throughout the country, inciting interest in associated research activities, which contributed largely to the prevention of stroke and hypertension.

By 1958, methods for screening of circulatory diseases and formats for their management had been introduced at academic meetings related to social medicine and the number of those who reported on the results of these screening tests increased. This trends for patient management spread to the congresses on clinical medicine, resulting in increases in the number of reports on sequential studies, such as early diagnosis of stroke and cardiac diseases, their clinical courses and prognosis. This trend led to the establishment of the Japanese Association for Cerebro-cardiovascular Disease Control, the activities of which encompassed not only the treatment but also a search for the etiology and prevention of the disease. If one looks back on the history of the reports presented at academic congresses, this was somewhat unique. Screening employees for circulatory diseases in industrial organizations was that for a huge mass that continued to grow in size in association with rapid economic growth, which led to health management and prevention. The improvement in health education and the work environment subsequent to screening later yielded notable results.

## STUDY SESSIONS AT SCIENTIFIC MEETINGS

In April 1958, a symposium entitled “Epidemiology of Hypertension” was held at a congress of the Japanese Society for Hygiene^53^ and in October of the same year, the Japanese Society of Public Health hosted a symposium on hypertension.^54^ The subjects of the major presentations at these meetings are introduced below as those reflecting the actual status of research at that time.

At the congress of the Japanese Society for Hygiene, the following presentations and discussions, with extensive and far-reaching content, were introduced: On the variability of blood pressure (Akira Nukada); Epidemiology of hypertension (Fumio Komatsu); Epidemiologic study of hypertension (Naosuke Sasaki); Relationship between excessive intake of silicates from the water supply and food and hypertension (Takayoshi Misawa); Regional differences in age-related changes in blood pressure (Eiji Takahashi); sex- and area-related differences in hypertension (Taeko Morooka); Regional differences in hypertension (Shoshi Tanaka); Regional clustering of stroke mortality (Yoshikatsu Nose); Correlation between acidity of water and stroke mortality (Jun Kobayashi); and Heredity of hypertension (Sadanobu Miyao).

At the Japanese Society of Public Health, the focus was on studies of the methods used for mass screening of circulatory diseases (including blood pressure monitoring, by Section on Adult Health). In spite of insufficient research support, studies were advanced to a certain level of sophistication. The presentations are listed below: Mass screening of blood pressure (Gunma); Blood pressure level in a work group (Tohoku, Miyagi); Studies on stroke, blood pressure and life style in an apple-producing area (Aomori and Akita); Blood pressure, obesity and body types in agricultural villages (Gunma); Blood pressure and proteinuria (Gunma); Blood pressure survey in upland farming area (Saitama); Blood pressure management in farming villages (Saitama); Fundus occuli findings and mass screening of hypertension (Chiba); Detailed examination of hypertension in employees of a government agency (Tokyo); Survey on blood pressure of employees in a public corporation (Tokyo); Health screening of Tokyo Municipal Government employees (Tokyo); Survey on blood pressure and electrocardiographic findings among factory employees (Tokyo); Screening of the circulatory system among police employees (Tokyo); Screening of employees of a pharmaceutical manufacturer (Tokyo); Improvement in dietary habits of hypertensives (Tokyo); Care of the circulatory system at homes for the aged (Tokyo); Health survey on the aged and hypertension (Yokohama); Statistical observation of blood pressure in an agricultural area (Niigata); Variability in hypertension (Nagano); Survey on hypertension in agricultural villages (Aichi); Survey conducted in agricultural villages (Shiga); Survey on circulatory diseases in agricultural villages in Osaka: blood pressure screening of general citizens (Osaka); Screening the circulatory system of the entire population of an agricultural village (Joint International Study, Fukuoka); Statistics on circulatory diseases seen in the department of internal medicine of a university hospital (Kurume); and Screening of hypertension: 2 titles (Nagasaki University). As evident in the listing, the subject matter was numerous and diverse.

Concerning the care of circulatory diseases seen in multiphasic health screenings (“Human Dock”), the following are listed: Follow-up survey of the patients examined at multiphasic screenings (Tokyo); Survey on the actual status of adult diseases (Yamanashi); Mass screening for hypertension: fundus examination (Nagano); Survey methods at multiphasic screenings (Aichi); Seasonal changes in blood pressure (Nagoya City); and Surveys at multiphasic screenings, blood pressure determination, ECG and fundus oculography (Mie). There were a large number of presentations; and judging by the discussions, the care of the circulatory system was becoming commonplace throughout the country through the efforts of regional services and business corporations. It should be noted that at this congress, Noboru Kimura, a pioneer in clinico-epidemiologic studies of cerebrovascular diseases, delivered a special lecture entitled “Moot Points in Etiological Factors for Vascular Diseases.”

Screenings of the cardiovascular diseases for regional residents and at the place of employment were further expanded after 1960 because gainful results could be obtained by following-up with a regional studies of a group of around 2,000 adult individuals; and thanks to financial support from the area, studies could be conducted even without economic resources. However, the studies were conducted mainly by clinical physicians with the participation of a number of industrial physicians, while epidemiologic specialists were rarely listed. On the other hand, a number of physicians in administrative positions (such as those at public health clinics) and other related personnel participated in these studies because the program was an important work at the respective area. Already by 1959, a group for the study of “Health Management of Patients with Hypertension or Cardiac Diseases” (chairperson: Shigeo Okinaka) was organized with a grant for Health and Science Research awarded by the Ministry of Health and Welfare and clinico-epidemiologic study sessions (including the methodology) were initiated.

At the general meeting of the 16th Congress of the Japanese Association of Medical Sciences held in 1959, a symposium on “hypertension” was held with Professor Fusakichi Nakazawa presiding as chairperson.^56^ The following studies were presented: “Pathogenesis of hypertension (Magojiro Maekawa); Hemodynamics, neurological modulation and other effects in hypertension (Kuju Saito); followed by epidemiologic study topics by Naosuke Sasaki in “Mass and individual evaluation of hypertension”; “Socio-medical factors in the onset of hypertension” (Shigetaka Obuchi); “Epidemiologic study of hypertension in the southwestern area of Japan: Kyushu Region” (Noboru Kimura); “Epidemiologic study of hypertension in the northeastern area of Japan?Tohoku region” (Katsuya Itahara); “Prognosis for mortality from hypertension” (Shizuo Torii); “Epidemiologic study of hypertensive ocular changes” (Akira Nakajima); and “Additional findings on etiology and treatment of hypertension” (Hideo Ueda). Sasaki exhibited the significance of blood pressure in a group and its relation to individual pressures and referred to the evaluation of blood pressure in each patient. Obuchi proved the participation of various environmental factors, in addition to individual constitutions, in the development of both stroke and hypertension. He analyzed the difference in the involvement of environmental factors (stratified by cities and agricultural villages) by using factor analysis and indicated a direction for its prevention based on the experimental results. Kimura introduced an epidemiologic study on stroke and cardiac diseases that had been conducted in several areas of Kyushu since 1956. He outlined its design and initial results. This was a cohort study that was conducted for a long time and its results appeared to be promising. Itahara conducted a comparative study of blood pressure distribution, characteristics of the hypertensives and nutritional state found in Akita, Hokkaido (on those who migrated from Akita), Miyagi and Nagano, to indicate the implication of the effect of environmental factors. Torii conducted a survey on 2,120,000 life insurance policy holders and analyzed a total of 38,102 hypertensive patients. Based on the results of statistical analyses, he set the criteria for blood pressure as follows: normal systolic pressure, 100 to 139 mmHg; borderline hypertension, 140 to 149 mmHg; and hypertension as 150 mmHg or over and he followed up the consequences. He observed changes in blood pressure due to aging. Nakajima described methods of classification of fundus findings, detection of abnormalities and key points in the procedure. Ueda described differentiation of diseases causing hypertension and their prognosis and referred to therapeutic approaches. All these are important findings as a basis for epidemiologic studies on circulatory diseases. One gets the impression from these studies that the precision in methodology for epidemiologic research had been raised to new heights.

While the clinical studies on stroke had not been completed, epidemiologic study intended for prevention and the practice of prevention itself have already taken place throughout the country, which was an unusual course for studies on diseases. Around this time (during the first half of the 1950s), frequency distributions and characteristics of blood pressures were discussed in a number of studies in which systolic and diastolic pressures were set at 150 and 90 or 100 mmHg, respectively. Since then, however, a new standard -- 140 and 90 mmHg for the systolic and diastolic pressures, respectively -- came to replace the former.

At the First Congress of the Japan Geriatrics Society held in 1961, a symposium entitled “On hypertension” was held^3)^ , where Akira Nukada, Tokuro Fukuda and Fusao Akiyama introduced their work from the viewpoint of epidemiology. In the same year, the Ministry of Health and Welfare created the “Conference and Liaison Organization for the Prevention of Chronic Diseases.” Now one might say that we moved into an age of adult diseases. Around this time, the 40- to 60- year-old population numbered about 18 million. In 1960, an international symposium on hypertension was held.

With infectious diseases now under control, the Ministry of Health and Welfare shifted its focus to cerebrovascular diseases. In 1961 and 1962, it conducted nationwide surveys on the actual status of adult diseases to determine the magnitude of the problem.

## ACHIEVEMENTS OF THE CLINICO-EPIDEMIOLOGIC RESEARCH GROUP (OKINAKA GROUP)^56^^,^^57^

From the clinical aspect, on the other hand, Goldberg and Kurland in 1962 made an international comparison on mortalities due to cerebrovascular disorders. According to this study, the ratio of cerebral hemorrhage to cerebral infarction among Japanese was 12.5 to 1, which differed greatly from comparative foreign figures. This is an exceptional event in international comparisons and a question arose as to the diagnostic validity of these two disease entities. Already in 1958 in the United States, classification and diagnostic criteria for cerebrovascular disorders had been made public and re-evaluation of the diagnosis and classification of the causes of death had started, while clinical studies on stroke and hypertension in Japan were delayed. Facing this criticism, a group on the study of stroke (chairperson: Shigeo Okinaka) was initiated financed by a Scientific Research Fund from the Ministry of Health and Welfare; and an interdisciplinary study by clinicians, pathologists and epidemiologists, an unusual project in Japan, was initiated. With the cooperation of 23 scholars specializing in this field, a 3-year followup study was conducted on 20,621 inhabitants (9,896 males and 10,725 females) in 17 districts scattered throughout the country. They obtained blood pressure distributions and other data in a preliminary study to determine the diagnostic criteria for death due to stroke and stroke classification, then observed the pattern of development of cerebrovascular diseases and compiled the data. During the 3 years, there were 456 deaths due to cerebrovascular disorders or 35.8% of all mortalities. The ratio of cerebral hemorrhage to cerebral infarction as causes of death was 1.37: 1, which was lower than expected. However, for those between 40 and 60 years, the incidence of cerebral hemorrhage was twice as high as that of cerebral infarction; among those in their 70s, the incidences of the two clinical entities were almost equal; and among those over 80 years, cerebral infarction occurred more frequently. Among the hospitalized patients, cerebral infarctions occurred more often. In other words, Goldberg et al were correct in pointing out the weakness in differentiating these two diseases and an actual condition that is unique to this country was uncovered. The incidence of cerebrovascular disorders was 0.9% of the entire population group. This study group remained active for 3 more years and in 1969, the results of the 5-year follow-up study were published. Indicated was that mortality due to cerebrovascular disorders was 34.7% (735 patients) of the entire mortality and for cardiac diseases it was 15.1% (320 patients). The mortality due to cerebral hemorrhage was on a plateau but for cerebral infarction it had increased. The ratio of cerebral hemorrhage to cerebral infarction as causes of death was 1:1 (when the data were limited to the latter half of the study) and 1.22:1 when computed for the 5-year period. This study group conducted a patho-anatomical study and a basic clinical pathological examination of cerebrovascular disorders. After 1976, a detailed report was published in book form.^58^^,^^59^ Related items also reported in this study were: in mortality due to cerebrovascular disorders in 750 cases at homes for the aged, the ratio of cerebral hemorrhage to cerebral infarction as the direct cause of death was 1:0.6, the former dominating the latter according to the clinical diagnosis. On the other hand, the ratio is reversed (1:1.6) if one examines the indirect causes of death. In a study at a hospital that was manned by a specialist, the ratio of hemorrhage to infarction according to the clinical diagnosis did not differ much from other countries’ reports. Apart from the above-noted study, Tachio Kobayashi in 1962 evaluated 28,612 cases included in a nationwide survey on adult diseases (30 years or older), analyzed 218 who succumbed to stroke, and reported that blood pressure was proportional to mortality. In a follow-up study at an area where the mortality due to cerebrovascular disorders was very high (327 per 100,000), it was reported that mortalities from cerebral hemorrhage, cerebral embolism and cerebral infarction were 7, 6, 3, respectively, with that for hemorrhage not being particularly high.

As epidemiologists, Naosuke Sasaki and Istuzo Shigematsu of the Department of Epidemiology, National Institute of Public Health participated in this study. Epidemiologists were involved in the studies at Tanushimaru and Ushibuka towns (site of Kurume University) and at Hisayama town (Kyushu University), which constituted long-term clinico-epidemiologic projects.

Starting in 1961, Katsuki and his colleagues of the Second Department of Internal Medicine, Kyushu University started a circulatory and clinico-neurological examination on inhabitants over 40 years of age at Hisayama town (population: approximately 6,500), Fukuoka Prefecture with a followup 6 months later.^59^^-^^60^ In this program, a totally new research design was adopted: briefly, if a cerebrovascular disease develops in a resident, a member of the research group, together with the attending physician, participated in the diagnosis and treatment; after the initial examination, the team continued home visiting offering guidance; with some exception, all who succumbed were subjected to autopsy to confirm the cause of death; and a cross-sectional survey was repeated every 2 years, succeeded by follow-up studies. Clinico-epidemiologic study was started with clinicians at the helm and data -- such as the prevalence of cerebrovascular diseases, their morbidity, prevalence and severity of hypertension, annual changes and electrocardiographic findings -- were accumulated. In fact, 10 years later, the cause of death was confirmed in 81.4% of all who had succumbed and the actual status of cerebral hemorrhage, embolism, infarction, subarachnoid hemorrhage and other diseases was examined in minute detail. Such clinical conditions as therapeutic effects, identification of the risk groups and followup of late consequences were analyzed. Through these observations, etiological factors and directions for preventive policies were revealed. It was a prospective study, producing reliable data, thus attracting the attention of many medical researchers. The study offered evidence for the increasing incidence of cerebral infarction in this country and new findings, such as the actual status of TIA were amassed.

Such a joint study triggered discussions on the classification and diagnostic criteria of cerebrovascular disorders, methods of blood pressure determination, accuracy of the test values obtained from area groups, all of which resulted in improved research accuracy. The importance of epidemiologic studies (such as the screening of local residents) gradually came to be understood by general practitioners. At that time, epidemiologists were rare in Japan but many clinical physicians participated in epidemiologic studies, while physicians assigned to local public health centers also joined in the project, representing the local legislative body. This was important in the development of epidemiologic studies. Some of the clinical physicians later became specialists in epidemiology and researchers in hygiene and public health served to expand the realm of epidemiologic studies. Young physicians were attracted to work under epidemiologists. However, research funds allocated to projects related to epidemiology were rather limited and there were few official positions in this area. Thus epidemiology could not develop as rapidly as one might wish.

## INTERNATIONAL SYMPOSIA AND RESEARCH DEVELOPMENT

The main theme of the ABCC symposium held in Hiroshima in 1966 was “Epidemiology of Coronary Heart Disease and Cerebral Apoplexy”. A report was made on the comprehensive research that had been conducted in Japan and the United States.^62^^-^^63^ The results of the follow-up studies on the atomic bomb-exposed patient group conducted by ABCC and those in the early stage of the prospective studies -- such as the resident-based epidemiologic surveys on cardiac diseases and stroke, which were started at Ushifuka and Hisayama towns of Kyushu -- were introduced and attracted attention. The clinico-eidemiologic study of cardiac diseases, part of the NI-HON-SAN research of the United States in which Japan participated, was introduced. It was the first international congress for epidemiologic and preventive medicine to be held in Japan after the end of the war.

For reports related to stroke at this symposium, an outline of an epidemiologic and clinicopathological study of 3 years that was conducted by the aforementioned Okinaka group was introduced. This outline included, as stated earlier, the following: in Japan, the incidence of cerebral hemorrhage had been high but that of cerebral infarction had been low but recently the latter was increasing in facilities for the aged; mortality and blood pressure were directly related; mortality was high in those with electrocardiographic abnormalities; and the occurrence of brain death generally appeared to be on a decline and death due to cardiac diseases was gradually increasing. Related to these topics, Itsuzo Shigematsu, an epidemiologist, compiled an interim report from the observations of blood pressure and nutritional state through a 2-year follow-up survey on about 1,700 subjects in their 50s and residing in 5 Prefectures. For the results of a 3-year follow-up survey on about 1,600 subjects residing in 3 areas in Kyushu, Noboru Kimura reported on a correlation among blood pressure, skin thickness, serum cholesterol level, nutrient intake and the incidence of stroke. Yasuo Hirota introduced an outline of a follow-up research plan for resident care (described later) at Hisayama town, Kyushu. Katsuhiko Yano of ABCC reported on a follow-up of atomic bomb victims. His report included the following findings: when stroke data were analyzed, the incidence of cerebral infarction was higher than that of cerebral hemorrhage; stroke incidence was high when the systolic pressure was over 160 mmHg and diastolic pressure over 90 mmHg; high incidences of stroke were also associated with electrocardiographic abnormalities, heart enlargement, diabetes mellitus (in women) and proteinuria. He also reported that a greater risk for stroke was associated with lower educational status (in both men and women), high meat consumption and a low intake of dairy products. At the end of the symposium, a discussion took place on preventive measures such as guidance of the patient after stroke, current status of therapy and instructions on diet surveys.

At this conference, the epidemiology of ischemic heart diseases that occurred frequently in the United States was discussed on the previous day and Japanese hospital surveys and the results of resident-level surveys conducted in various parts of Japan were introduced. Study reports from Tokyo, Yamanashi, Nagoya/Gifu, Kyoto, and Kyushu and also from the atomic bomb victims’ organization were introduced but they are omitted here.

## SUBSEQUENT RESEARCH REPORTS

Many of the studies at industrial organizations were continually reported. The number of individuals in the groups was limited and the subjects were not likely to migrate: if screening and management were thorough, the effects of intervention would become evident shortly. In the Tokai region, large and middle-sized industrial organizations joined forces to form a study group to manage tuberculosis and conducted research and discussions. Around 1960, the evaluation of methodology and effects, in addition to screening and management of the circulatory system, came to be discussed.^51^ This type of organization spread throughout the country and an academic meeting was held each year.^52^ Many research bodies were organized. Thus within 10 years, the mortality due to circulatory diseases among the employees of these organizations was halved in comparison with that of ordinary citizens.

The achievement of Fukuda et al^64^ compiled in book form as the results of work of many years, is introduced here as a model of an epidemiologic study of circulatory diseases conducted by business organizations. Around 1960, Fukuda started the care of the circulatory system for the national railway system, based around Tokyo. Starting in 1965 when screening results were stabilized, he compiled the results of 9 years of observations on 230,000 subjects over the age of 45 years. The basic population was large and unlikely to migrate and both the survey method and the follow-up were based on almost perfectly homogenous data. Accurate grasp of the incidence within the group was truly epoch-making. The research encompassed almost every conceivable item relative to the subjects: blood pressure fluctuation and health management, effect of health guidance, effect of daily living, changes in the frequency of stroke and heart attacks, blood pressure level, physical status, health management and guidance, effect of life style, relationship between the development of circulatory diseases and various factors such as obesity, somatic type, serum cholesterol level, ECG findings, environmental conditions, work conditions, effects of antihypertensive treatment, and epidemiologic observation of acute cardiac death and uremia. The work had a significant influence not only on subsequent epidemiologic studies but also on clinical medicine. It is indeed an epidemiologic study with preventive significance that can be proudly presented to an international audience. Lastly, a discussion is presented on how this significant effect of circulatory care at the work site should be utilized in the management of farmers threatened by high stroke mortality.

## SUBSEQUENT STUDIES ON ETIOLOGICAL FACTORS

The resident-based cohort study at Hisayama town has been continued, and good quality of data have been accumulated. The research results concerning such topics as etiological surveys, therapeutic approaches and preventive measures were highly promising.

The NI-HON-SAN study, which started in 1965 with the cooperation of the United States and Japan, also played an important role. It is a prospective study on Japanese-American and native Japanese males (residing in Hiroshima and Nagasaki) designed for a comparative evaluation of the development of circulatory diseases and their etiologies in two different environments where they resided.^65^^-^^66^ These subjects were composed of 3,000 men residing in Hiroshima and born between 1895 and 1924 and 9,878 Japanese-American men from Hawaii. At Hiroshima and Nagasaki, the population-based disease registry started in 1957. This study was the best in controlling the racial factor. After 5 years, the total mortality among the Japanese in Hawaii was 2/3 that of the Japanese in Japan. Mortality due to stroke among Japanese was more than twice, while that due to cardiac diseases was only 1/2 of those in Hawaii. The incidence of cerebral hemorrhage was about the same in the two groups but cerebral infarction occurred twice as often and cerebral arteriosclerosis 3 times more often among the Japanese in Japan. These findings proved that the regional difference in the incidence of circulatory diseases was mostly due to environmental factors. For the etiological factors in Japanese in Japan, a low protein and fat (in particular of animal origin) intake and consumption of large quantities of carbohydrates, alcohol, and sodium were cited. There was no significant difference in blood pressure, which had been considered to be problematic or slightly higher among the Japanese Americans. The incidence of ischemic heart diseases was also somewhat higher among the Japanese in Hawaii. In other words, a greater number of preventable environmental factors were detected more clearly, which is a great advantage of epidemiologic studies. The effects of labor conditions were also indicated. This study has been continued since this report, offering many new findings and serving as a source of information for designing preventive measures. Since 1965, reliable prevalence and morbidity rates have been obtained in Hiroshima and Nagasaki, thus fulfilling the contents of epidemiologic studies.

## DEVELOPMENT OF STUDIED AMONG LOCAL RESIDENTS IN JAPAN

For epidemiologic studies of circulatory diseases, several years of follow-up surveys even in a small area with a population of around 20,000 produced reasonably satisfactory results. Therefore even in an age when budgets were stringent, such a study was possible when clinical physicians and epidemiologists cooperated. Since 1970, larger research grants from the National Government became available, more substantial epidemiologic surveys became possible and studies on preventive methods were conducted in earnest. The mass screening format was studied and evaluated from the early days by specialists in social medicine, resulting in the establishment of highly reliable screening formats. Small electrocardiograms were developed in the latter half of the 1960s. Improved through experimental use in various places, the type that could withstand use at actual mass screenings finally became available on the market in the 1970s. With the availability of this electrocardiogram, mass screening for circulatory diseases will become even more available in this country.

The study of cerebrovascular diseases developed in parallel with progress in clinical study, pathology and epidemiology. Supported by research in the biochemical field, it progressed ahead of epidemiologic studies on non-infectious diseases in post-war Japan. On the other hand, in several agricultural villages in this country, joint efforts by enthusiastic clinicians and local legislative bodies enabled low-profile but steady continuation of care and prevention for circulatory diseases for residents, producing a notable effect on the management of stroke and hypertension.

## ESTABLISHMENT OF THE JAPANESE ASSOCIATION FOR CEREBRO-CARDIOVASCULAR DISEASE CONTROL

In 1959, the Conference on the Management of Chronic Diseases was founded, with the Osaka Adult Disease Center (at present; Osaka Medical Center for Cancer and Cardiovascular Diseases) at its core; and in 1965, the Japanese Association for Cerebro-cardiovascular Disease Control was organized. Its establishment was agreed upon at the free meeting on hypertension of the 22nd Congress of the Japanese Society of Public Health for comprehensive research of circulatory diseases, with the cooperation of clinicians and epidemiologic and public health specialists for organic coalition among researchers, the establishment and dissemination of various types of standards and wide application of various policies. The organization was first established by nominating Tachio Kobayashi as chief director, 15 leaders in the field of clinical medicine and epidemiology as administrative officers and 2 as inspectors. In 1968, it was approved as an incorporate association. The membership was around 750 in 1962, representing diverse fields such as clinical medicine, epidemiology-public health, and industrial management. With this group at its helm, evaluation of research methods, technical assistance, training, hosting research meetings and health education of local residents were provided.

Sasaki^3^ prepared a detailed report on the state of research around the time of the Conference and research meetings.

## DEVELOPMENT OF STUDIES ON CIRCULATORY DISEASES

In 1968, World Health Organization (WHO) published Cardiovascular Survey Methods co-authored by Rose GA and Blackburn H,^68^ which set out the standards for such items as survey plans, preparation, methods for medical history taking, physical testing and special tests. It subsequently became an international textbook for the examination of the circulatory system. Also the 1970 WHO Conference Report, “WHO Cerebrovascular Diseases: Prevention, Treatment and Rehabilitation Report”^69^ set the standard for diagnosis at an international level; and research was conducted based on these criteria. In our country, an accurate morbidity and incidence of cerebrovascular diseases had not been determined (except at Hiroshima) and the time was ripe for preparing basic data from Japan that would be suitable for international comparison.

In 1969, the Ministry of Health and Welfare introduced a special program to prevent stroke, aiming to halve the stroke mortality: it named model cities, towns and villages, appealed to the late middle aged population to seek examinations, encouraged therapeutic management and health guidance and further improved mobile health management units and screening centers located in agricultural villages. Already by 1961 and 1962, the Ministry had conducted basic surveys on adult diseases; and based on the outcome of this survey, it estimated the number of hypertensive patients over the age of 40 to be 8.07 million. Thus the Welfare Law for the Aged was instituted and a system for health examinations of those over 65 years of age was organized. In 1971, a program for physical rehabilitation was initiated. The number of patients seeking treatment for cerebrovascular diseases tripled in the 10-year period after 1963 and for those with cardiac diseases it doubled. Thus the country needed strongly positive legislative measures.

Since 1970, the local legislative bodies in those legislative areas with high mortalities for circulatory diseases actively invited circulatory examinations; and study teams and clinical physicians cooperated with epidemiologic researchers to advance these examinations and studies. Throughout the country, the numbers of research projects on epidemiology and prevention of circulatory diseases increased. In the 1970s, a research commission with Yoshio Komachi, an epidemiologist, acting as leader, and funded by the Ministry of Health and Welfare, was started to undertake a nationwide joint study for circulatory diseases including heart disease. Meanwhile a comprehensive international study comprised of 7 countries and guided by Keys of the United States was initiated. Noboru Kimura of Japan participated in its activities. In 1970, WHO introduced a guideline concerning the standards for measuring blood pressure and methods of examination. Epidemiologic studies and prevention measures were started at a level acceptable the world over.
